# Women’s persistent depressive and perinatal grief symptoms following a miscarriage: the role of childlessness and satisfaction with healthcare services

**DOI:** 10.1007/s00737-017-0742-9

**Published:** 2017-06-16

**Authors:** Francine deMontigny, Chantal Verdon, Sophie Meunier, Diane Dubeau

**Affiliations:** 1 Université du Québec en Outaouais, PO Box 1250, Hull Station, Gatineau, QC J8X 3X7 Canada; 2 Université du Québec à Montréal, Montreal, Québec Canada

**Keywords:** Miscarriage, Depression, Perinatal grief, Childlessness, Satisfaction with healthcare services

## Abstract

The objectives of this cross-sectional study were to determine whether depressive and perinatal grief symptoms vary according to time since miscarriage and to test whether childlessness and satisfaction with healthcare services influence symptom duration. A total of 245 women who had experienced a miscarriage answered a self-report questionnaire, indicating the date of their miscarriage and assessing their present level of depressive and perinatal grief symptoms. They also provided sociodemographic characteristics and indicated their level of satisfaction with healthcare services. One-way analyses of variance indicated that women who had miscarried within the past 6 months reported higher scores for depressive symptoms than did women who had miscarried between 7 and 12 months ago and more than 2 years ago. However, when controlling for childlessness and satisfaction with healthcare services, those differences became respectively marginal and non-significant, indicating that depressive symptoms are similar across time for more than 2 years after the loss. Regarding perinatal grief, results revealed that symptoms significantly decreased across time only for women with children and women who were satisfied with healthcare services. For childless women and those dissatisfied with healthcare services, perinatal grief symptoms did not vary according to time since miscarriage. Results suggest that, particularly for women who are childless and/or dissatisfied with healthcare services, depressive and perinatal grief symptoms persist long after a miscarriage. These results highlight the importance of paying particular attention to more vulnerable women and of improving healthcare services post-miscarriage.

## Introduction

Miscarriage, defined as the premature loss of an embryo or a fetus within the first 27 weeks of pregnancy (Klier et al. 2002), is an important and prevalent event. Indeed, about 15% of all clinically recognized pregnancies end in miscarriage (Hemminki and Forssas 1999; Kline et al. 1989). Many studies have reported the deleterious impact of miscarriage on women’s mental health (Beutel et al. 1995; Klier et al. 2002; Neugebauer et al. 1992; Neugebauer et al. 1997). However, results concerning the duration of depressive and perinatal grief symptoms following a miscarriage are inconsistent. Indeed, some studies have indicated that, for most women, symptoms resorb and are similar to those of the general population within 6 months to 1 year (Brier 2008; Broen et al. 2006; Janssen et al. 1996; Cumming et al. 2007; Lok et al. 2010), while others have indicated that depressive symptoms persist long after the loss, for up to 3 years (Beutel et al. 1995; Blackmore et al. 2011). These results raise the question, why do some women recover more rapidly than others from the psychological burden of a miscarriage? The aim of the present study was to provide some answers to this question by examining whether depressive and perinatal grief symptoms vary according to time since miscarriage, and whether childlessness and satisfaction with healthcare services can influence the association between depressive and perinatal grief symptoms and time since miscarriage.

## Miscarriage and women’s mental health

There is increasing recognition that miscarriage can seriously affect women’s mental health. In fact, miscarriage, usually seen as the death of an expected child, is considered a traumatic event (Klier et al. 2002). Empirical studies have reported elevated depressive symptoms among women in the months following a miscarriage (Beutel et al. 1995; Lok et al. 2010; Neugebauer et al. 1992). Moreover, studies have found that 10 to 15% of women who experience a miscarriage attain the clinical threshold for a major depressive disorder in the months following the event (Beutel et al. 1995; Cumming et al. 2007; Neugebauer et al. 1997).

In addition to depressive symptoms, after a miscarriage, women may experience perinatal grief and have symptoms of bereavement, such as emotional numbness and a yearning for the lost child (Beutel et al. 1995; Lee and Rowlands 2015). Indeed, miscarriage is comparable to other forms of loss in terms of intensity and duration (Brier 2008). Even though grief and depression share some similarities, they can be assessed and experienced separately. As pointed out by Lee and Rowlands (2015), following a miscarriage, some women may report grief symptoms, such as struggling with difficult emotions and trying to find meaning, while at the same time not experiencing depressive symptoms and being able to function well enough in everyday life. To date, however, most studies on miscarriage have focused on symptoms of anxiety and depression, and few have examined both depression and perinatal grief at the same time (see Beutel et al. 1995 for an exception).

## Duration of depressive and perinatal grief symptoms following a miscarriage

Results concerning the duration of depressive and perinatal grief symptoms following a miscarriage are inconsistent. Some studies have found that, for most women, depressive and perinatal grief symptoms resorb within 6 months to 1 year following the miscarriage (Brier 2008; Broen et al. 2006; Janssen et al. 1996; Cumming et al. 2007; Lok et al. 2010), while others have found that the deleterious effects of miscarriage on women’s mental health persist long after the loss. For example, Blackmore et al. (2011) found that depression and anxiety could persist up to 3 years after the miscarriage. In the same vein, Beutel et al. (1995) found that, while grief symptoms appear to last around 6 months, anxious and depressive symptoms persisted more than 1 year past the loss. Those results partly contradict what Lee and Rowlands (2015) found when integrating the results of both quantitative and qualitative studies, which was that some women might not experience long-lasting depressive symptoms yet still grieve for their loss many years following a miscarriage. Thus, results concerning the duration of depressive and perinatal grief symptoms following a miscarriage are inconsistent. Also, very few studies have exceeded the 1-year time frame usually applied in longitudinal studies examining the association between miscarriage and mental health, and little is known about its long-term impact.

Moderating variables have been identified to explain the inconsistent results concerning the association between mental health and time since miscarriage. For example, Lok et al. (2010) found that depressive symptoms persist 1 year after the loss only among women who were initially more distressed. Rowlands and Lee (2010a) also found that previous medically diagnosed depression or anxiety was associated with downward mental health trajectories across time among women who had miscarried. In addition to prior mental health, childlessness and satisfaction with healthcare services could also be important to consider when explaining why depressive and perinatal grief symptoms persist longer after a miscarriage for some women.

Childlessness has consistently been associated with depressive and perinatal grief symptoms after a miscarriage (Janssen et al. 1997; Neugebauer et al. 1992, 1997). Indeed, women with one or more living children have been found to be less at risk of developing depression (Neugebauer et al. 1997) or perinatal grief symptoms (Janssen et al. 1997) following a miscarriage. Along those lines, studies have examined whether a subsequent pregnancy and the birth of a healthy child might reduce depressive and grief symptoms related to a prior miscarriage. Blackmore et al. (2011) found that, among women with prior prenatal loss, depressive symptoms during a subsequent pregnancy persisted even after the birth of a healthy child. However, other studies have reported that a subsequent pregnancy is associated with a significant decrease in grief level (Cuisinier et al. 1996; Nikcevic et al. 1999). Brier (2008) suggests that a subsequent pregnancy enables women to retrieve important roles they had lost with the miscarriage (e.g., role of pregnant woman and mother). Retrieving those roles might help them to recover more rapidly from the grief associated with their miscarriage. Based on the inconsistent results mentioned above, it is conceivable that the presence of living children might influence perinatal grief more than depressive symptoms, but this hypothesis would need to be tested.

Regarding satisfaction with healthcare services, studies have indicated that support from health professionals has a positive impact on women’s experience of a miscarriage (Rowlands and Lee 2010b) and that women who were satisfied with their physician reported higher mental health scores (Rowlands and Lee 2010a). Despite the recognized importance of quality of health care following a miscarriage, studies continue to report that a majority of women are dissatisfied with the care they received (Rowlands and Lee 2010b; Simmons et al. 2006; Warner et al. 2012). Lack of empathy, compassion, information, and follow-up are identified as dissatisfaction factors (Geller et al. 2010). Further research is needed on the influence of satisfaction with healthcare services on women’s mental health following a miscarriage, including a variety of health services (e.g., nurses, midwives, psychologists), and on how satisfaction levels might explain the greater persistence of depressive and grief symptoms among some women.

## Study objectives

Results from previous studies highlight the need to examine the duration of depressive and perinatal grief symptoms following a miscarriage and to identify what factors might help some women recover faster than others from this experience. The first objective of the present study was to examine the level of depressive and perinatal grief symptoms following a miscarriage according to time since miscarriage. To add to existing studies, time since miscarriage was extended to include more than 2 years after the loss. The second objective was to examine whether the presence of children and satisfaction with healthcare services can influence the association between time since miscarriage and depressive and perinatal grief symptoms. The aim was to gain a better understanding of the conditions under which perinatal grief and depressive symptoms might resorb faster and to identify areas of vulnerability where attention is needed to mitigate the deleterious effect of miscarriage on women’s mental health.

## Methods

### Procedure and participants

A cross-sectional study was conducted in the province of Quebec, Canada. Women were recruited through ads posted on conventional and social media. Ads were also placed in medical clinics and emergency rooms and conveyed in bereavement support groups, and participants were referred from health professionals working in those settings. Women interested in participating called a research assistant, who verified whether they met the inclusion criteria: (1) aged 18 years and older; (2) having experienced at least one miscarriage in the past 6 years; and (3) being able to read French. Eligible participants could answer the self-report questionnaire either by phone with the research assistant, or on their own, on a secured web platform. A total of 245 women answered the questionnaire, all of whom chose to use the online version.

### Measures

#### Depression

The French version of the Edinburgh Postnatal Depression Scale (Cox et al. 1987; Guedeney and Fermanian 1998) was used to assess depressive symptoms. This ten-item scale shows good face, criterion, and construct validity (Guedeney and Fermanian 1998). Its reliability has also been demonstrated with satisfactory internal consistency (*α* = 0.76) and high test–retest reliability (ICC = 0.91) (Guedeney and Fermanian 1998). Respondents are asked to indicate how they have felt over the past 7 days, using a 4-point Likert scale ranging from 0 to 3. Scores for all the questions are summed to obtain a global score ranging from 0 to 30 (*α* = 0.89 in the present study). A score of 10 or higher is usually used as a cutoff for possible depression (Cox et al. 1987).

#### Perinatal grief

The French version of the Perinatal Grief Scale—Short Version (Potvin et al. 1989; de Tychey and Dollander 2000) was used to measure perinatal grief symptoms. This 33-item scale shows good psychometric characteristics (de Tychey and Dollander 2000). Respondents are asked to indicate how they presently feel regarding their miscarriage (e.g., *I am grieving for the baby*). Reversed scores were recoded, and items were summed to produce a global perinatal grief score (*α* = 0.94 in the present study). A score >91 is considered high (Toedter et al. 2001).

#### Satisfaction with healthcare services

A 19-item scale was created to assess women’s satisfaction with healthcare services. Participants were asked to indicate how helpful they perceived a list of 19 professional health services received during their miscarriage (nurse, physician, gynecologist) or afterward (support groups) on a 4-point Likert scale (1 = not helpful; 2 = somewhat helpful; 3 = helpful; 4 = very helpful). Participants could also indicate “not applicable” if they had not received the service during their miscarriage or afterward. Scores for each service received were averaged to obtain a global satisfaction score. Participants with a mean score ≥3 were classified as satisfied, with the remainder classified as dissatisfied.

#### Sociodemographic variables and childlessness

Participants were asked to indicate their age, household income, highest education level, and country of birth (Canada/other). They were also asked to indicate the date of their latest miscarriage. Time since miscarriage was categorized as less than 7 months, between 7 and 12 months, between 1 and 2 years, and more than 2 years. Participants were asked to indicate the number of living children they presently had. A dichotomous variable was created: 1 = childless and 0 = with children.

### Statistical analysis

First, missing data were calculated. Since they were minimal (less than 1%), nothing was done to replace them and listwise deletion was used for main analysis. Then, descriptive statistics were obtained. The number of participants in each category was calculated for categorical variables, while mean and standard deviation were calculated for depression and perinatal grief continuous scores. One-way analyses of variance (ANOVA) were conducted to examine whether depressive and perinatal grief symptoms varied by time since miscarriage. When a significant result was found (*p* < .05), Tukey’s post hoc tests were performed to investigate where the difference was positioned in relation to the time points. Finally, to test the influence of childlessness and satisfaction with healthcare services on the association between depressive and perinatal grief symptoms and time since miscarriage, 2 (childlessness or satisfaction with healthcare services) × 4 (time since miscarriage) ANOVAs were performed. When a significant interaction was found, file splitting was used and ANOVAs were performed to examine whether depressive and perinatal grief symptoms varied according to time since miscarriage at different levels of childlessness and/or satisfaction with healthcare services. To control for family-wise alpha error rate while testing for those two simple effects for each analysis, statistical significance was lowered to *p* < .025.

## Results

Table 1 presents participants’ characteristics. Mean age was 31 years, with a majority of participants between 25 and 34 years. Most participants were born in Canada, more than three quarters had a household income of at least 50,000 CAD and most had a college (technical or pre-university) or university degree. Participants were equally distributed by time since miscarriage, with about one quarter of the sample in each category. Participants in each of those categories did not differ significantly in terms of sociodemographic characteristics (age, education, income, country of birth). Finally, more than one third of participants were childless and almost one third were dissatisfied with the healthcare services they received.

**Table 1:** Participants’ characteristics

	Number (%)^a^
Age (years)	
18–24	21 (8.57)
25–34	167 (68.16)
≥35	55 (22.45)
Family income (CAD)	
0–49,999	54 (22.04)
50,000–99,999	108 (44.08)
≥ 100,000	83 (33.88)
Education	
High school	39 (15.92)
College (technical or pre-university)	72 (29.39)
University	130 (53.06)
Country of birth	
Outside of Canada	17 (6.94)
Canada	228 (93.06)
Time since miscarriage	
0–6 months	65 (26.53)
7–12 months	58 (23.67)
1–2 years	64 (26.51)
>2 years	58 (23.67)
Childlessness	
Yes	92 (37.55)
No	153 (62.45)
Satisfaction with healthcare services	
Satisfied	160 (65.31)
Dissatisfied	78 (31.84)

^a^Percentage ratios are based on the total *N* (245)

Table 2 presents mean scores for depression and perinatal grief symptoms by time since miscarriage. One-way ANOVAs indicated significant differences in depressive symptoms (*F*_(3, 241)_ = 3.24, *p* = .023) according to time since miscarriage. More specifically, Tukey’s post hoc tests indicated that women who had miscarried in the past 6 months had a higher score for depressive symptoms than did women who had miscarried between 7 and 12 months ago or more than 2 years ago. However, the score for depressive symptoms among women who had miscarried between 1 and 2 years ago was similar to that of women who had miscarried within the past 6 months. As for perinatal grief, results indicated no significant difference in mean score according to time since miscarriage (*F*_(3, 241)_ = 2.49, *p* = .061).

**Table 2:** Depression and perinatal grief symptoms by time since miscarriage

	Time since miscarriage				*F*	*p*
	0–6 months	7–12 months	1–2 years	>2 years		
Depressive symptoms Mean (SD)	12.62 (5.67)	9.66 (6.51)	10.97 (6.37)	9.57 (6.45)	3.24	.02
Perinatal grief symptoms Mean (SD)	78.49 (26.60)	68.88 (30.22)	73.45 (31.90)	65.02 (26.48)	2.49	.06

To examine the influence of childlessness on the association between time since miscarriage and women’s depressive and perinatal grief symptoms, 4 (time since miscarriage) × 2 (childlessness yes/no) ANOVAs were conducted. Regarding depressive symptoms, results revealed that the main effect of childlessness (*F*_(1, 237)_ = 8.60, *p* = .004) was significant. Childless women had higher scores for depressive symptoms than did women who had children (*M* = 12.24 vs. 9.88). The main effect of time since miscarriage was marginally significant (*F*_(3, 237)_ = 2.63, *p* = .051) while the interaction between time since miscarriage and childlessness (*F*_(3, 237)_ = 2.22, *p* = .087) was not.

**Fig. 1:**
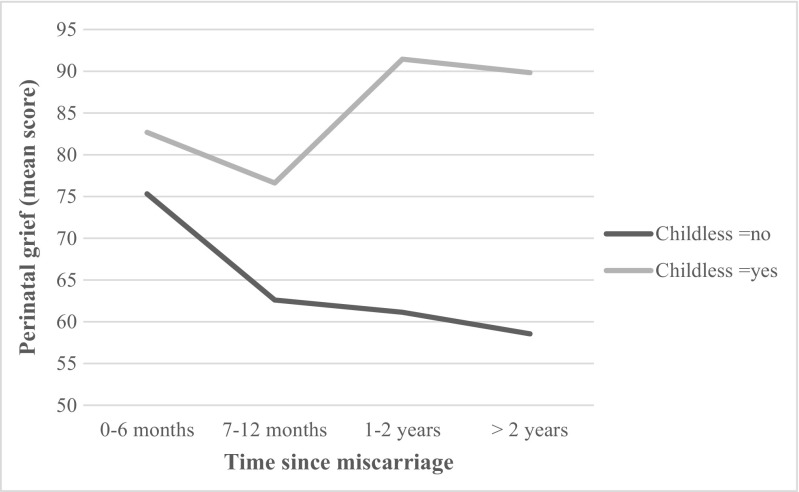
Perinatal grief symptoms as a function of time since miscarriage and of childlessness

Similarly, for perinatal grief, the main effect of childlessness was significant (*F*_(1, 237)_ = 31.00, *p* < .001), while the main effect of time since miscarriage was not (*F*_(3, 237)_ = 1.28, *p* = .282). Childless women had a higher score for perinatal grief symptoms (*M* = 84.38 vs. 64.09) than did women with children. The interaction between time since miscarriage and childlessness was significant (*F*_(3, 237)_ = 2.68, *p* = .047). As illustrated in Fig. 1, for women with children, perinatal grief symptoms decreased by time since miscarriage, while for childless women, perinatal grief symptoms increased and remained stable. Indeed, decomposition of main effects revealed that, for childless women, perinatal grief levels did not vary according to time since miscarriage (*F*_(3, 88)_ = 1.11, *p* = .348). However, there was a significant difference among women with children (*F*_(3, 149)_ = 3.70, *p* = .013), where women who had miscarried more than 2 years ago (*M* = 75.32, SD = 26.33) reported significantly fewer perinatal grief symptoms than did women who had miscarried within the past 6 months (*M* = 58.54, SD = 22.11).

**Fig. 2:**
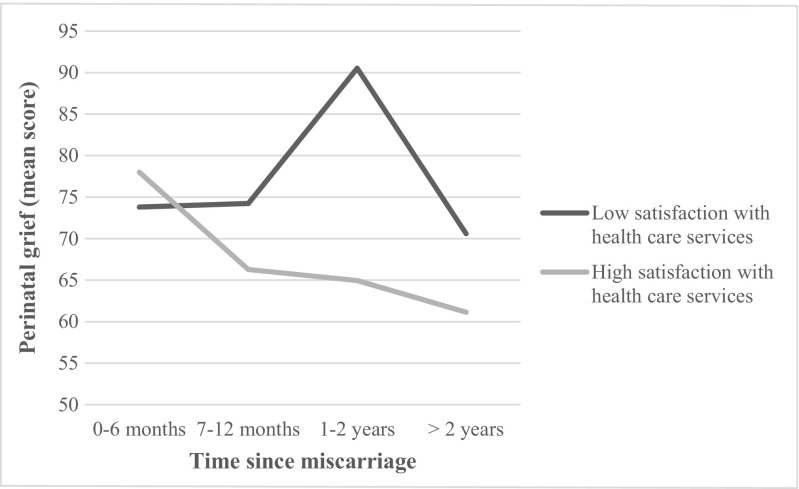
Perinatal grief score as function of time since miscarriage and satisfaction with healthcare services

Finally, to examine the influence of satisfaction with healthcare services on the association between time since miscarriage and women’s depressive and perinatal grief symptoms, 4 (time since miscarriage) × 2 (satisfaction with healthcare services yes/no) ANOVAs were conducted. Results revealed that, for depressive symptoms, neither the main effects of time since miscarriage (*F*_(3, 230)_ = 2.22, *p* = .086) and of satisfaction with healthcare services (*F*_(1, 230)_ = 3.05, *p* = .082) nor the interaction of those two variables (*F*_(3, 230)_ = 2.01, *p* = .114) were significant. As for perinatal grief, the main effect of time since miscarriage was not significant when included with satisfaction with care in the eq. (*F*_(3, 230)_ = 1.59, *p* = .193), while the main effect of satisfaction with care was significant (*F*_(1, 230)_ = 7.32, *p* = .007). Women who were satisfied with healthcare services had lower scores for perinatal grief symptoms (*M* = 67.75, SD = 26.14) than did women who were not (*M* = 77.88, SD = 31.74). The interaction between time since miscarriage and satisfaction with healthcare services was marginally significant (*F*_(3, 230)_ = 2.63, *p* = .051). As illustrated in Fig. 2, among women who were satisfied with healthcare services, perinatal grief symptoms decreased with time. In contrast, among women who were dissatisfied with healthcare services, there was an increase in perinatal grief symptoms for those who had miscarried between 1 and 2 years ago. Decomposition of main effects revealed that, for women who were dissatisfied with healthcare services, there was no difference in perinatal grief symptoms according to time since miscarriage (*F*_(3, 74)_ = 1.35, *p* = .265). However, there was a significant difference among women who were satisfied with healthcare services (*F*_(3, 156)_ = 3.31, *p* = .022). Specifically, there was a significant difference between women who had miscarried more than 2 years ago (*M* = 61.28 SD = 20.28) and women who had miscarried in the past 6 months (*M* = 78.00 SD = 26.43).

## Discussion

The purpose of the present study was to examine post-miscarriage depressive and perinatal grief symptoms according to time since miscarriage. Results indicated higher scores of depressive symptoms among women who had recently miscarried, with those who had miscarried within the past 6 months having higher scores for depressive symptoms than those who had miscarried between 7 and 12 months ago or more than 2 years ago. At first glance, these results are in line with those of other studies showing a significant reduction of depressive symptoms between 6 months and 1 year after miscarriage (Brier 2008; Broen et al. 2006; Cumming et al. 2007; Janssen et al. 1996; Lok et al. 2010). However, over the longer term, it can be seen that women who had miscarried between 1 and 2 years ago continued to report high levels of depressive symptoms and did not significantly differ from women who had miscarried within the past 6 months. Hence, while depressive symptoms scores were lower among women who had miscarried between 7 and 12 months ago, there was an apparent upsurge in symptoms among women who had miscarried between 1 and 2 years ago. Difficulties conceiving another child, anniversaries, or other significant dates associated with the miscarriage might explain this upsurge in depressive symptoms. However, further analysis indicated that when controlling for childlessness and satisfaction with healthcare services, the effect of time since miscarriage on depressive symptoms became respectively marginally and non-significant, indicating that those symptoms remain stable, even long after the miscarriage. Also, with regard to perinatal grief, there was no significant reduction in symptoms for up to more than 2 years after the miscarriage. In sum, the results of the present study converge with those of Blackmore et al. (2011) and, to some extent, of Beutel et al. (1995) in finding that depressive and perinatal grief symptoms can persist long after the loss, for up to 3 years past the miscarriage.

The second objective of the study was to identify factors that might explain why depressive and perinatal grief symptoms might persist longer after a miscarriage. Childlessness was identified as one such factor. Indeed, for childless women, there was no significant difference in perinatal grief symptoms according to time since miscarriage. For those women, grief symptoms remained stable regardless of time since miscarriage, and those who had miscarried more than 2 years ago showed levels of perinatal grief similar to those of women who had recently miscarried. In contrast, for women with children, perinatal grief symptoms were significantly lower among women who had miscarried more than 2 years ago. These results are in line with those of other studies reporting that women with living children are at lower risk of depression following a miscarriage (Janssen et al. 1997; Neugebauer et al. 1997) and that grief symptoms decreased after the subsequent birth of a healthy child (Cuisinier et al. 1996; Nikcevic et al. 1999). However, it was not possible in the present study to determine whether the children were born before or after the miscarriage. Future studies should try to distinguish between those two situations and discern their respective impacts on depressive and perinatal grief symptoms. Nevertheless, childless women appear to be more vulnerable to the persistence of grief symptoms long after a miscarriage. In a randomized control trial evaluating the efficacy of a supportive counseling program for women who had miscarried, Kong et al. (2014) found that not all women benefited from the program and noted that women who were more vulnerable (i.e., women with high levels of psychological distress) should be targeted. In this vein, results of the present study indicate that health professionals should pay particular attention to childless women and follow up with them on a long-term basis.

Nonetheless, it should be noted that in the present study, childlessness did not influence the association between depressive symptoms and time since miscarriage. This result is similar to that of Blackmore et al. (2011), who found that, among women who had miscarried, depressive symptoms remained stable after the subsequent birth of a healthy child. Thus, childlessness appears to be more useful to explain the duration of perinatal grief rather than depressive symptoms.

Satisfaction with healthcare services also emerged as an important factor in the association between perinatal grief symptoms and time since miscarriage. For women who were dissatisfied with their healthcare services, there were no differences in perinatal grief symptoms across time: women who had miscarried more than 2 years ago showed levels of perinatal grief symptoms similar to those of women who had miscarried more recently. In contrast, for women who were satisfied with the healthcare services they received, levels of perinatal grief symptoms decreased by time since miscarriage and were significantly lower among women who had miscarried more than 2 years ago. These results strongly suggest that the quality of healthcare services for women going through a miscarriage might directly influence their mental health in the following months and even years. It should be noted, however, that the measure of satisfaction with healthcare services was rather heterogeneous, with participants only assessing healthcare services they received during and after their miscarriage (indicating not applicable for other services). It is therefore not possible to identify which health services (i.e., nurses, physicians, support groups, etc.) are more likely to contribute to women’s satisfaction. Future studies could assess satisfaction with healthcare more precisely by focusing on one type of service at a time. Nevertheless, studies to date have reported that women are generally mostly dissatisfied with the care they received before, during, and after their miscarriage (Rowlands and Lee 2010b; Simmons et al. 2006; Warner et al. 2012), indicating that there is much to be done to assess women’s needs and tailor healthcare services accordingly. Early pregnancy assessment clinics (EPAC), successfully implemented since 1991 in the UK and, more recently, in Australia, New Zealand, and Canada (Tunde-Byass and Cheung 2009; Rhone et al. 2012), appear to be an innovative solution to improve women’s satisfaction with services. In an EPAC, specialized personnel (physician and nurse) support women in the natural, medical, or surgical resolution of their miscarriage. An evaluation conducted in Australia showed that EPAC clinics reduced emergency wait times by 55%, repeat visits for the same problem by 48%, and request for laboratory tests by 56% (O’Rourke and Wood 2009). Evaluations of Canadian EPAC clinics have obtained similar results, also confirming client satisfaction (Tunde-Byass and Cheung 2009; Rhone et al. 2012). This care model is thus interesting in terms of improving quality of care and reducing health system costs and should be considered for wider deployment.

Finally, as with childlessness, satisfaction with healthcare services did not influence the association between time since miscarriage and depressive symptoms. Future studies should focus on other variables that could explain under what circumstances depressive symptoms are more likely to resorb faster. For instance, previous history of depression is strongly associated with postpartum depression (Neugebauer et al. 1997) and is probably an important variable to consider. However, it was not assessed in the present study. The use of a more precise measure of depression, such as the Structured Clinical Interview for DSM IV (SCID interview), could have given important information about the history of depression (e.g., number of episodes, chronicity) and would be recommended for future studies. Another limitation of the present study concerns the cross-sectional design, which precludes any cause-and-effect conclusions. Also, since a convenience sample was used, it is possible that women with higher levels of depressive and perinatal grief symptoms following their miscarriage were more drawn to the present study and volunteered to participate, even if their miscarriage had happened a long time ago. Notwithstanding this recruitment bias, the results of the present study are consistent with those of studies with large numbers of participants (Blackmore et al. 2011; Rowlands and Lee 2010a, 2010b). Finally, it should be noted that the question assessing childlessness did not specify whether the other children, if any, were born before or after the miscarriage (for women who had miscarried more than 1 year ago). Thus, it is not possible to know whether it was the presence of a living child before the miscarriage or the birth of a subsequent healthy child, or even both, that influenced the duration of depressive and perinatal grief symptoms.

## Conclusion

Miscarriage has important repercussions on women’s mental health. The present study indicates that depressive and perinatal grief symptoms can persist long after the loss. Results also revealed that perinatal grief symptoms significantly decreased across time only for women with children and women who were satisfied with healthcare services. For childless women and those dissatisfied with healthcare services, perinatal grief symptoms did not vary according to time since miscarriage and were stable for more than 2 years after the loss. Those results were not replicated with depressive symptoms, since childlessness and satisfaction with healthcare services did not influence symptom reduction across time. Other relevant variables, such as mental health history, should be taken into account in future studies. Nevertheless, these results highlight the need to optimize healthcare services offered to women before, during, and after a miscarriage. They also emphasize the importance of paying particular attention to vulnerable women and offering them support on a long-term basis.
